# Rice breeding for low input agriculture

**DOI:** 10.3389/fpls.2024.1408356

**Published:** 2024-06-21

**Authors:** Subroto Das Jyoti, Gurjeet Singh, Anjan Kumar Pradhan, Lee Tarpley, Endang M. Septiningsih, Shyamal K. Talukder

**Affiliations:** ^1^ Department of Soil and Crop Sciences, Texas A&M University, College Station, TX, United States; ^2^ Texas A&M AgriLife Research Center, Beaumont, TX, United States

**Keywords:** sustainability, high-throughput phenotyping, genomics, genomic selection, organic rice, low-input agriculture

## Abstract

A low-input-based farming system can reduce the adverse effects of modern agriculture through proper utilization of natural resources. Modern varieties often need to improve in low-input settings since they are not adapted to these systems. In addition, rice is one of the most widely cultivated crops worldwide. Enhancing rice performance under a low input system will significantly reduce the environmental concerns related to rice cultivation. Traits that help rice to maintain yield performance under minimum inputs like seedling vigor, appropriate root architecture for nutrient use efficiency should be incorporated into varieties for low input systems through integrated breeding approaches. Genes or QTLs controlling nutrient uptake, nutrient assimilation, nutrient remobilization, and root morphology need to be properly incorporated into the rice breeding pipeline. Also, genes/QTLs controlling suitable rice cultivars for sustainable farming. Since several variables influence performance under low input conditions, conventional breeding techniques make it challenging to work on many traits. However, recent advances in omics technologies have created enormous opportunities for rapidly improving multiple characteristics. This review highlights current research on features pertinent to low-input agriculture and provides an overview of alternative genomics-based breeding strategies for enhancing genetic gain in rice suitable for low-input farming practices.

## 1 Introduction

Agriculture is one of the survival factors for humans on this planet. However, our intensive agriculture practices severely endangered our climate. Agriculture and related activities emitted 9.3 billion tons of CO_2_ eq. in 2018 (FAO, 2020). Additionally, the agriculture sector contributed around 9% of the US greenhouse gas emission and was the largest supplier of the US N_2_O emission in 2021 (EPA, 2023). Notably, fertilizer application aiming for higher nitrogen availability was the reason for higher N_2_O emission, and the contribution of urea fertilization in CO_2_ emission was 5.2 MMT CO_2_ eq. (EPA, 2023). The extensive use of chemical fertilizers and pesticides also harms the ecosystem by contaminating groundwater and other natural resources. Although these practices ensure higher yields, they are destroying our environment.

Thus, we need a transition to a sustainable as well as low-input (LI) agriculture system to avoid further environmental problems in the future.

We can plan a sustainable agriculture system by ensuring economic profitability, a healthy environment, and social development while safeguarding our natural resources (Horrigan et al., 2002). One way to build a sustainable agriculture system is to modify our modern farming systems into low-input systems. For instance, researchers described that low-input systems could lead to sustainable agriculture by achieving efficiency in conventional practices (Hill and MacRae, 1996). A profitable yield can be achieved with lower fertilizer input with appropriate nutrient management (Good and Beatty, 2011; Chen et al., 2014). Popular high-yielding varieties require a constant supply of synthetic fertilizers and pesticide. For this reason, the success of these LI systems depends on the development of high yielding cultivars adapted to low-input conditions. Further, Evans (1996) stated that the success of any cropping system depends on the synergistic interaction between all inputs, such as fertilizers, irrigation, weed control, etc. Therefore, agronomic practices that ensure positive interaction between all these inputs will be crucial for LI systems.

Rice is one of the most extensively consumed cereals globally. It is the number one staple food among developing countries and also the most vulnerable crop to climate change (Wassmann et al., 2010). Since a large portion of world population consume rice, rice production needs to be increased to ensure food security across the globe. Further, the global population is forecasted to cross 9 billion by 2050 and more than 10 billion by 2080 (UN, 2022). For this reason, maintaining rice acreage across the globe is very important. However, extensive use of chemical fertilizers and pesticides significantly contributes to agricultural pollution. In this context, growing rice cultivars suited for LI systems is vital in practicing sustainable agriculture.

However, the most challenging part of developing crop cultivars for the LI system is incorporating tolerance to various biotic and abiotic factors, nutrient use efficiency, and higher yields. Therefore, rice breeders should target multiple traits to develop extremely resource-efficient cultivars. From the era of the green revolution, several genotypes were characterized with specific traits through conventional breeding approaches. The genomics era has opened many windows for rice breeders to understand the function of different pathways with traits of interest, which directly support rice improvement. It is challenging for rice breeders to combine all the traits into single cultivars, thus integrated breeding approaches help to develop multiple-traits cultivars (Singh et al., 2024). Fortunately, modern genomics tools increased the efficacy of multi-trait breeding and reduced the time required for integrating numerous traits. Besides, the low-cost genotyping techniques have enabled plant breeders to decipher multiple characteristics of a population in a cheaper and faster way. Speed breeding can be incorporated with various modern breeding tools in numerous steps of a breeding pipeline to reduce the breeding cycle and enhance selection accuracy and efficiency (Gudi et al., 2022). However, more effort must be made to include all the modern genomic tools to develop varieties suitable for LI agriculture. As far as we know, none of the suitable articles are available discussing the LI system for sustainable agriculture regarding the breeding perspectives. Keeping this in mind, our goal is to prepare a precise review on rice breeding for the LI system. Hence, we reviewed the requisite traits and genomics-based strategies for breeding rice for a low-input agriculture system.

## 2 Environmental impact and need for LI in rice production system

**Figure 1:**
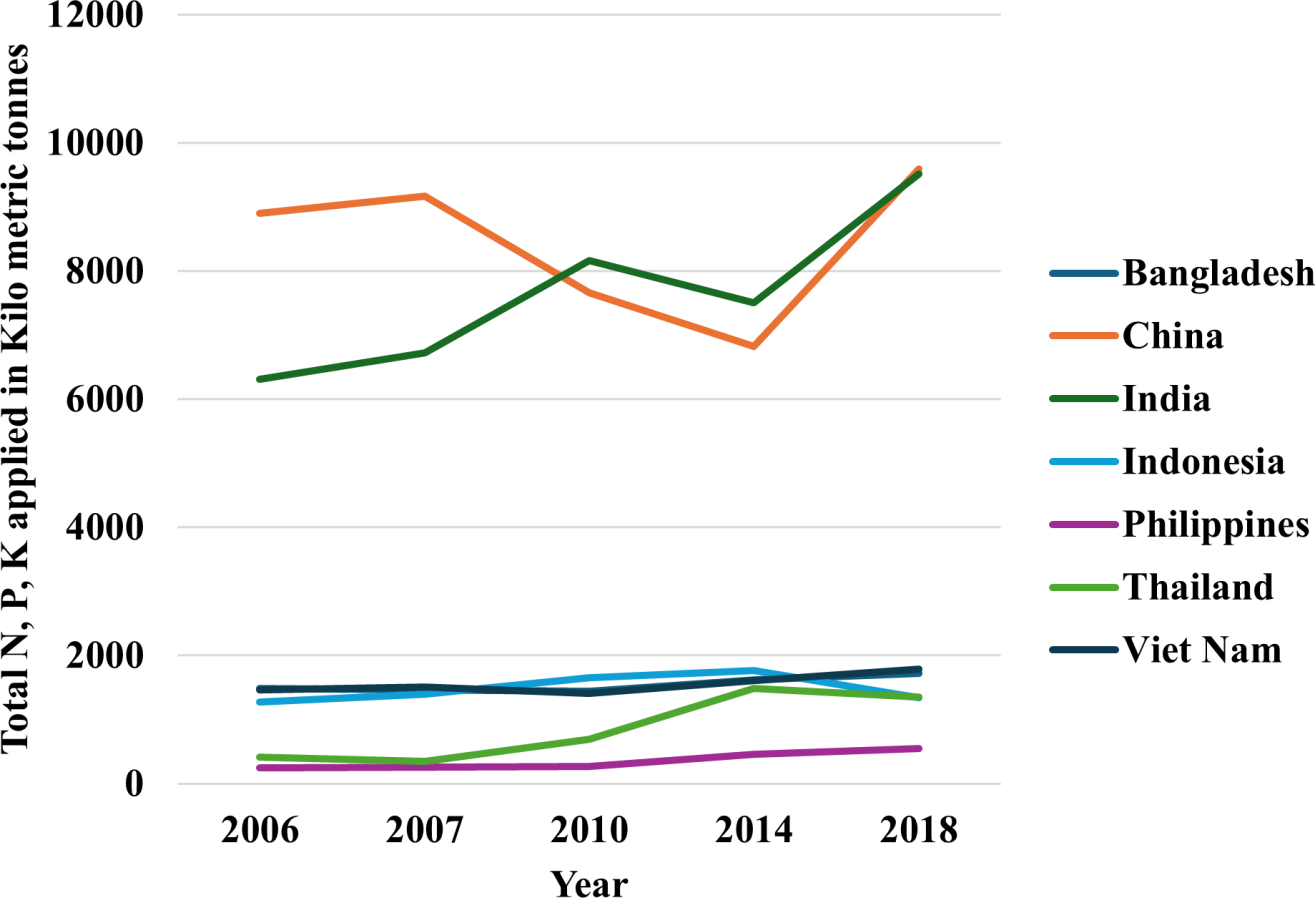
Total N, P_2_O_5_, K_2_O fertilizer used in rice per year in top rice producing countries over the last two dacades (Ludemann et al. 2022a). The dataset used for this figure can be found in Ludemann et al. (2022b).

Following the “green revolution,” chemical fertilizer and pesticide use in modern agriculture has increased tremendously worldwide (**Figure 1**). Although high-input systems ensure rapid fiscal growth, these systems damage the environment. High-input rice cultivation is practiced around the globe to feed the massive population. Rice has higher greenhouse gas emission potential than other major cereals. For example, rice has a 467% higher GWP (Global Warming Potential) than wheat and 169% higher GWP than maize (Linquist et al., 2012). Rice fields are significant contributors to CH_4_ and N_2_0. Previous data suggested that rice fields are responsible for 30% and 11% of world agricultural CH_4_ and N_2_O emissions, respectively (IPCC, 2007). For this extensive rice cultivation, carbon dioxide and other greenhouse gases are increasing quickly, endangering all living beings on Earth.

Furthermore, the heavy dosage of pesticides and fertilizers exert residual effects on foods, creating carcinogenic impact on the consumer’s body. Besides, pesticides contaminate natural resources like water and alter the harmony between many biological processes. Excessive input is closely related to the environmental damage caused by rice cultivation (Ahmad et al., 2023). Therefore, an alternative low-input system is needed to safeguard our nature while sustaining agricultural growth.

Efficient use of fertilizer and exploring plant’s inherent resource use efficiency will reduce greenhouse gas emissions and remove the risk of contamination from chemical pesticides. A previous study reported that low input farming will help in better management of soil fertility in the long run. Since LI farming increases the amount of organic C and stored nutrients in the soil, LI farming will gradually increase soil health (Clark et al., 1998). Management practices like crop rotation will establish proper nutrient cycling and preserve soil productivity. The LI system will also indirectly positively affect biodiversity by minimizing water and air pollution. Moreover, the LI system is the most feasible remedy for the negative impact of conventional agriculture systems.

## 3 Low-input agriculture

The ongoing modern high-input agriculture system needs an alternate solution due to the fast-changing climate and various agricultural pollutions. Despite having wide cultivation areas across the globe, rice production can be intensified sustainably (Yuan et al., 2021). Though improved varieties are being developed worldwide, significant yield gaps exist in many rice cropping regions. Inefficient fertilizer use and lack of proper management are the prime causes of this discrepancy (Yuan et al., 2021). Moreover, it is essential to simultaneously increase both yield and resource use efficiency for a sustainable farming system. Breeding for better nutrient use efficiency will help to reduce the yield gap present in certain rice growing regions.

Some rice cropping systems have lower yields despite having higher N input (Yuan et al., 2021). In those systems, N (Nitrogen) input can be reduced while having higher yield by increasing crop resource use efficiency (Yuan et al., 2021). Therefore, the development of resource-use-efficient rice cultivars that can be cultivated in LI systems is recommended. Researchers began to emphasize the value of LI systems around the turn of the twenty-first century. These systems use the least production input while management procedures are upheld to guarantee a successful crop output (Parr et al., 2020). The primary focus of low-input systems is minimizing off-farm resources such as pesticides and fertilizer to reduce environmental pollution and improve soil health.

Additionally, LI system relies on on-farm resources like management practices to generate maximum yield output. Notably, LI systems offer a plethora of environmental benefits over conventional methods. For instance, the LI system ensures lower rates of N leaching and mineralization, which helps maintain future sustainability (Poudel et al., 2002). A study conducted for eight years with crop rotation with different crops, including rice, showed that low-input and organic farming improved soil chemical properties (Clark et al., 1998). Although organic farming has long been touted as a sustainable agriculture practice, it produces lower yield per acre than conventional farming (Seufert et al., 2012).

In contrast, the LI system can reduce the trade-offs between environmentally benign farming methods and financial success. Crops in LI systems must rely on effective resource utilization and innate defensive mechanisms since they get minimal external input. Consequently, LI systems yield less than traditional systems. Two significant issues that restrict profitability under low input systems are the lack of N supply and weed competition (Clark et al., 1999). Cultivars suited for low input circumstances should be designed considering multiple traits and a range of selection environments to address different yield-limiting issues. In summary, LI agriculture needs inclusions of two major factors for sustainable rice farming: selection of resource efficient crops and adaptation of proper management practices to minimize the wastage of natural resources. Initially, a goal might be set up for 20% resources reduction during rice crop management i.e. nitrogen and/or and water use, while maintaining similar or enhanced yield.

## 4 Breeding for low-input agriculture

**Figure 2:**
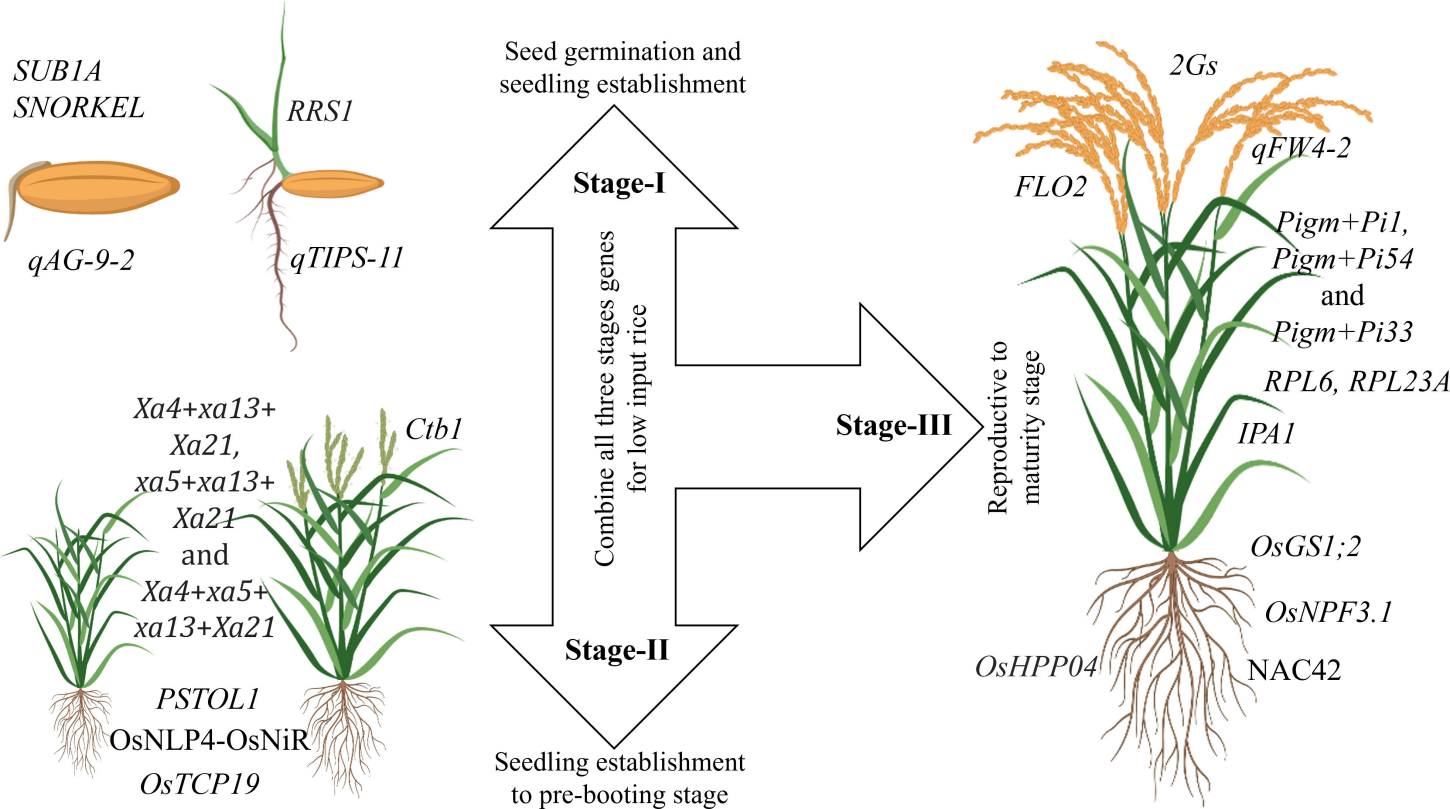
Important genes and QTLs necessary for developing ideal rice cultivar for LI systems. Stage-I: Germination and seedling vigor under various stress conditions; *SUB1A* gene confers tolerance to submergence, *SNORKEL* gene controls plant elongation to escape deepwater (Miro and Ismail, 2013), *qAG-9–2* is associated with tolerance to flooding during germination (Angaji et al., 2010), *qTIPS‐11* is associated with increased lateral root number(Wang et al., 2018a), and *RRS1* is a negative regulator of root development. Knockout of *RRS1* in plants enhances root growth, including longer root length, lateral root length, and higher lateral root density, drought resistance by promoting water absorption and improving water use efficiency (Gao et al., 2023). Stage-II: Vegetative to pre-booting stage; Effective gene combinations like *Xa4*+*xa13*+*Xa21, xa5*+*xa13*+*Xa21* and *Xa4*+*xa5*+ *xa13*+*Xa21* are widely utilized for bacterial blight resistance (Pradhan et al., 2020), OsNLP4-OsNiR is associated with increased tiller number and yield through enhancing nitrogen assimilation and nitrogen-use-efficiency (Yu et al., 2021), *OsTCP19* allele is closely associated with high tillering response to soil nitrogen (Liu et al., 2021), *PSTOL1* enhances grain yield in phosphorus-deficient soil and also acts as an enhancer of early root growth, thereby enabling the plants to acquire more phosphorus and other nutrients (Gamuyao et al., 2012), *Ctb1 is* associated with cold tolerance at the booting stage (Saito et al., 2010). Stage-III: Reproductive to maturity; *IPA1* controls the ideal plant architecture in rice and is associated with drought tolerance (Chen et al., 2023a); 2Gs genes influence grain weight and grain number i.e., *GW7* enhances grain weight) and *DEP2* grain number. Both are co-localized on chromosome 7. Thus, there is a possibility of simultaneous introgression for both grain number and weight improvement (Singh et al., 2024), *qFW4*-*2 is* associated with flag leaf size and photosynthetic capacity. *FLO2* plays a pivotal regulatory role in grain size and starch quality by affecting storage substance accumulation in the endosperm (She et al., 2010), *OsHPP04* is associated with resistance to root-knot nematode without any adverse effects on plant growth (Huang et al., 2023), Among the cloned genes, *Pigm+Pi1*, *Pigm+Pi54* and *Pigm+Pi33* are the most effective gene combination patterns to achieve the stable broad-spectrum resistance to both leaf blast and panicle blast under various conditions, these resistance gene combination patterns have potential in gene pyramiding breeding (Ning et al., 2020), OsNAC42 activates a haplotype of nitrate transporter *OsNPF6.1^HapB^* that confers high nitrogen use efficiency by increasing yield under low nitrogen supply (Tang et al., 2019), *OsGS1;2* and *OsNPF3.1* increase NUE (Hang et al., 2024), *RPL6* and *RPL23A* increase water use efficiency (Moin et al., 2016, 2017).

Plant characteristics are the decisive factor for adaptation in a particular environment. Similarly, the ability of the plant to survive in a pesticide-free and nutrient-scarce system is essential for LI system. Therefore, traits that improve nutrient-usage-efficiency, weed competency, and multiple stress tolerance are critical for LI systems. Breeding for ideal rice plant ensures the incorporation of important traits into a single genotype to develop cultivars for low-input rice. Genes that control seedling establishment, vigor and multiple stress tolerance must be utilized to enhance seedling establishment to the pre-booting stage (**Figure 2**). For better yield under LI systems, genes controlling grain yield and nutrient use efficiency should be explored and utilized (**Figure 2**).

### 4.1 NUE and PUE

Rice fields are heavily fertilized almost all over the world. An estimation of fertilizer use in rice across top rice producing countries have been depicted in **Figure 1**. For low-input systems, nitrogen (N) and phosphorus (P) are comparatively more crucial than other nutrients (Wolfe et al., 2008). Remobilizing leaf-stored nitrogen during grain filling is essential in increasing nitrogen use efficiency (Mickelson et al., 2003; Fan et al., 2007). In cereals, N remobilization accounts for a significant portion of grain N content (Kichey et al., 2007; Fan et al., 2020). Similarly, remobilizing stored P contributes to a substantial portion of phosphorus loading in grain (Julia et al., 2016). Previous studies suggested that pectin helps remobilize P (Zhu et al., 2015). However, the presence of nitrate interferes with the pectin synthesis and hinders P remobilization (Zhu et al., 2018). For these reasons, breeding for both nitrogen and phosphorus use efficiency is a challenging task.

### 4.2 Root traits

Researchers also emphasized the importance of root morphology for optimal nutrient acquisition, as roots are a vital participant in nutrient uptake. However, the N and P uptake requires opposing root types for maximum efficacy. In general, less lateral development and a deeper root structure with fewer axial roots are recommended for higher nitrogen usage efficiency. On the contrary, more axial root and lateral growth are needed for improved P and K efficiency (Lynch, 2022).During the past few years, numerous studies have been conducted on features linked to nitrogen use efficiency. Plants uptake nitrogen as a nitrate ion (Lynch, 2019). In addition, the “steep, cheap, and deep-rooted” ideotype has improved corn’s ability to collect nutrients in ideotype breeding (Lynch, 2013). In rice, two classes of lateral roots named L-types (long and thick) and S-type (short and small) are found (Yamauchi et al., 1987; Ajmera et al., 2022). Henceforth, increasing the fraction of nodal roots with smaller diameters, shallower nodal root angles, and large densities of L-type roots may boost yield potential in low nitrogen conditions (Ajmera et al., 2022). Conversely, P efficiency may be attained by increasing axial root production, shallower axial root development, root hair length, and root hair biomass (Lynch, 2019). Therefore, combining diverse ideotypes with root phenotypes can be a helpful strategy for breeding nutrient usage efficiency (Lynch, 2019). Nonetheless, the phenotyping of roots is tedious and requires much effort and time. For this reason, high-throughput phenotyping systems can be very advantageous in various breeding programs. Therefore, root phenotyping for better nutrient use efficiency can be easily achieved. For root phenotyping, image processing systems like GT-RootS (Borianne et al., 2018), DeepLabv3 (Shen et al., 2020), and ChronoRoot (Gaggion et al., 2021) were claimed to be helpful in high throughput phenotyping.

### 4.3 WUE

Rice cultivation heavily depends on the availability of water. Minimizing the water requirement in rice fields is beneficial from both the environmental and economic viewpoint. In general, WUE is measured by studying the leaf structure and gas exchange dynamics in leaves. Most researchers used the Carbon isotope discrimination (Δ13C) method to study WUE in rice. Lower Δ13C is an indication of higher WUE, and researchers located multiple QTLs governing WUE across the rice genome through this method ( (Xu et al., 2009; This et al., 2010; Roja et al., 2016).

Multiple genes related to WUE in rice have been identified. For example, *RPL6* and *RPL23A* are documented as target genes for increasing rice WUE (Moin et al., 2016, 2017). Another study highlighted the role of the *OsαCA1* gene in increasing rice WUE (He et al., 2023). Transgenic rice expressing *AtTOR* genes also showed higher WUE (Bakshi et al., 2017). In addition, the *BLANKET LEAF (BKL)* gene of *Oryza nivara* is also suggested as a potential target for increasing water use efficiency (Hamaoka et al., 2017). Interestingly, a study reported that aquaporin expression profiles in rice roots are also a significant determinant of WUE (Nada and Abogadallah, 2014).

Since there is no rapid and cost-effective method of measuring WUE, breeding for WUE is arduous and time-consuming. Meanwhile, management practices in rice field can be manipulated to ensure maximum WUE. For example, previous studies reported that alternate wetting drying could increase WUE in rice without hampering yield (de Avila et al., 2015; Wang et al., 2016, 2020a). Therefore, a modified crop management system with proper fertilization can be an excellent way to increase water use efficiency in rice (Xue et al., 2013).

### 4.4 Early vigor

Early vigor is another essential feature of the LI system. Early vigorous plants will have the requisite strength to compete against weeds. In rice, many studies identified traits that regulate early vigor in various conditions (Namuco et al., 2009). Traits such as specific leaf area, leaf area index, early tillering ability, root length, root density, time to maturity, and growth duration affect the weed competitiveness of rice (Dingkuhn et al., 1999; Fofana and Rauber, 2000; de Vida et al., 2006; Rao et al., 2007) also demonstrated that early vigor and light interception traits are essential for weed competitiveness in rice.

### 4.5 Others

Besides weeds, plants must fight invading pathogens from multiple sources. Since the LI input system will use no or low amount of pesticides, plants should have inherent defense capacity against various diseases. Luckily, the magnitude of soil-borne disease is much lower in the LI system due to better soil quality (van Bruggen et al., 2016). A recent study reported that rice-pulse rotation can improve the microbiome diversity and decrease the pathogen population in aerobic rice field ( (Panneerselvam et al., 2023). Yet, the crop must achieve resistance against other pathogens prevalent in LI systems. The stay-green trait is also essential for abiotic stress tolerance. For delayed senescence, the stay-green trait can provide prolonged photosynthesis, but reducing remobilization can hamper nitrogen use efficiency (Plett et al., 2017). UAV-based techniques can contribute to plant breeding through quick and efficient phenotyping capacity (Xie and Yang, 2020). Recently, sensor-based phenotyping has been practiced in many crops. For example, multiple sensor-based UAV systems were described for measuring crop canopy-related traits and environmental data in soybean and wheat (Bai et al., 2016). In rice, UAV-based phenotyping is also being used for assessing nutrient content and disease resistance (Lu et al., 2021a; Bai et al., 2023; Shaodan et al., 2023).

Furthermore, phenotyping only in the LI system will not provide sufficient material for an ideal LI system. A sustainable commercial breeding program for LI systems should combine performance data from both high-input and LI systems (Muellner et al., 2014). To identify candidate alleles for LI agriculture, we should compare the performance of specific alleles from each input level and select the superior lines (Atlin and Frey, 1989). Combining phenotyping and genotyping data can produce unique inferences on features, and these conclusions may serve as the basis for applying contemporary breeding tactics to modify plants targeting LI systems.

### 4.6 Genomics-based breeding for LI system

**Figure 3:**
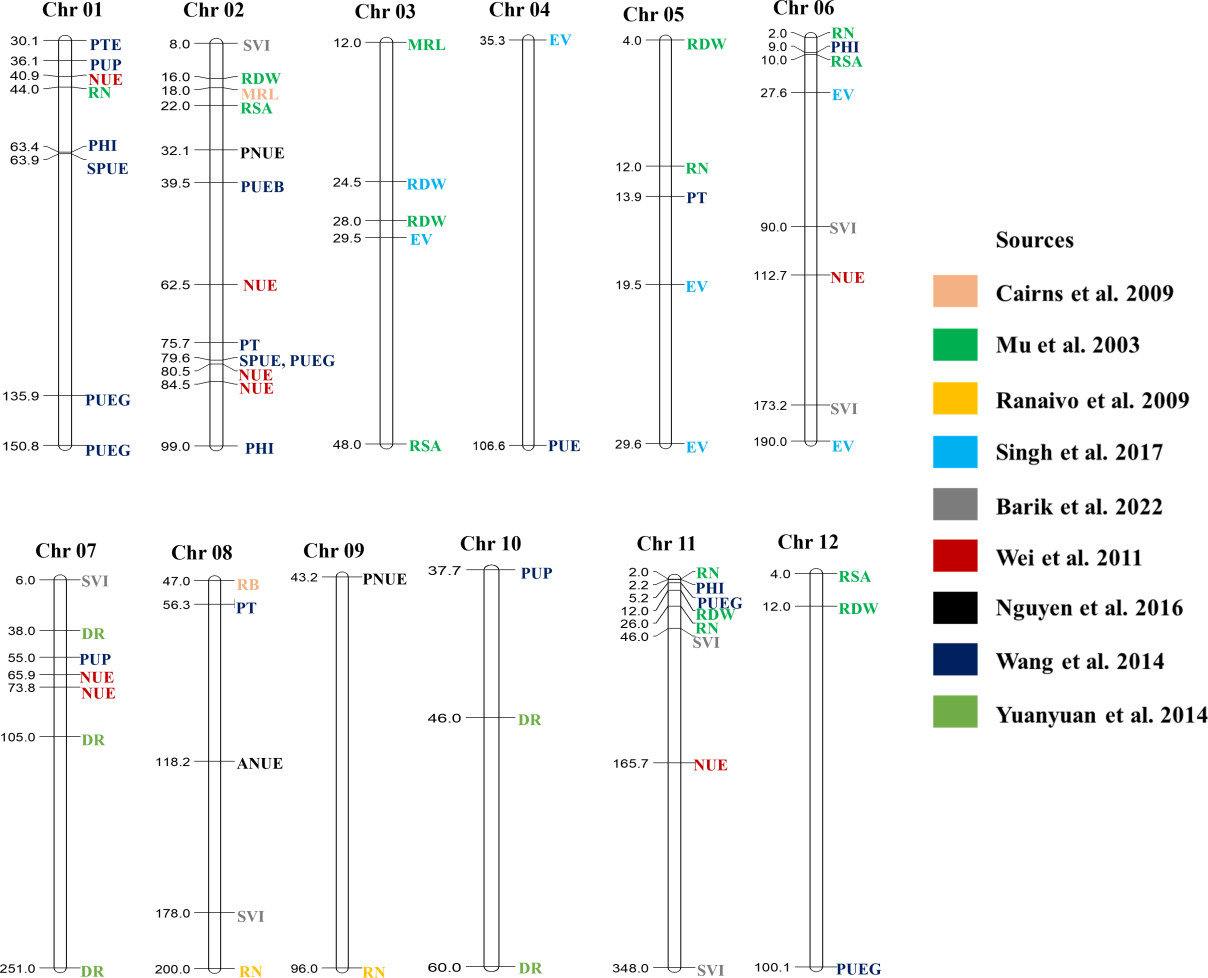
Reported QTLs in rice controlling traits related to LI systems. MRL, Maximum root length; RB, Root Biomass; DR, The number of deep roots; RSA, Root surface area; RN, The number of roots; RDW, Root dry weight; EV, Early vigor; NUE, Nitrogen use efficiency; PNUE, Physiological nitrogen use efficiency; ANUE, Agronomic nitrogen use efficiency; PTE, Phosphorus translocation efficiency; PUP, Phosphorus uptake at maturity; PHI, Phosphorus harvest index; SPUE, Straw phosphorus use efficiency; PUEG, Phosphorus use efficiency for grain yield; PUE, Phosphorus use efficiency; PUEB, Phosphorus use efficiency for biomass; PTE, Phosphorus translocation; SVI, Seedling vigor index; All genetic distance are in cM unit.

The spate of inexpensive sequencing techniques emerged as a blessing for plant breeding. Likewise, genotyping has become a routine task in breeding programs, and combining genotype and phenotyping data can provide critical information about specific traits of interest. As a result, characterizing and discovering new markers, genes, and QTLs for the desired phenotype has become more convenient nowadays. In addition, creating a sustainable agricultural system will require both the survival capacity of wild cultivars and superior agronomic traits from elite cultivars. Consequently, combining genes from wild and elite cultivars can be a way to develop sustainable cultivars (Dawson et al., 2008). The introgression of genes has been practiced in the pre-omic era through marker-assisted backcross breeding. Marker-assisted breeding for single genes from cultivated cultivars or marker-assisted backcross breeding for single genes from landraces has been practiced in different crops (Prasanna et al., 2020). Over the past few years, QTLs affecting characteristics essential for LI systems have also been explored (**Table 1**). Multiple regions of rice chromosomes have important QTLs related to resource use efficiency and seedling establishment (**Figure 3**). For example, QTLs related to nitrogen use efficiency, phosphorus uptake, phosphorus translocation, and root number have been reported between 30.1 to 44 cM region on chromosome 1 in rice (Wei et al., 2011; Wang et al., 2014). Phosphorus and nitrogen use efficiency related QTLs were also mapped between 75.7–84.5 cM region on chromosome 2 and 55–73.8 cM region on chromosome 7. On chromosome 11, QTLs controlling root traits and phosphorus use efficiency are located between the 2 to 5.2 cM region. However, most of the QTLs are polygenic in nature, thus it is challenging to enhance genetic gain in breeding improvement. This issue was resolved with the advent of genome-wide association studies (GWAS) that can detect numerous QTLs for more accurate documentation of allelic controls of the traits.

**Table 1:** Reported rice QTLs that control traits related to LI systems.

Traits	QTLs	Markers	Chromosome	Genetic position (cM)	Physical position (kb)	Conditions	Reference
Root length		OSR17	2	18			(Cairns et al., 2009)
	*qRL-1*	mk188-mk191	1		26,250–26,700		(Yang et al., 2021)
	*qRL-4*	mk963–mk964	4		13,450–13,550		
	*qRL-6*	mk1381–mk1382	6		6950–7150		
	*qRL-10*	mk2164–mk2165	10		2550–2650		
	*mrl2*	RM208-RM48	2	0			(Mu et al., 2003)
	*mrl3*	R2247-C746	3	12			
	*mrl8*	G187-RM310	8	0			
	*mrl5*	C282-R1838	5	0			
Root Biomass		RM284	8	47			(Cairns et al., 2009)
	*qRB3*		3	4.81			(Barnaby et al., 2022)
The number of deep roots	*qDR1.1*		1	184			(Yuanyuan et al., 2022)
	*qDR4.1*		4	187			
	*qDR5.1*		5	68			
	*qDR6.1*		6	130			
	*qDR7.1*		7	38			
	*qDR7.2*		7	251			
	*qDR7.3*		7	105			
	*qDR10.1*		10	46			
	*qDR10.2*		10	60			
Root Surface area	*qRSA-3*	mk758–mk763	3		24,550–25,050		(Yang et al., 2021)
	*qRSA-9*	mk2124–mk2127	9		20,850–21,150		
	*qRSA-11*	mk2505–mk2508	11		28,150–28,550		
Root diameter	*qRD-2*	mk302–mk303	2		650–750		(Yang et al., 2021)
	*qRD-8*	mk1915–mk1916	8		21,200–21,500		
Root Volume	*qRV-3*	mk758–mk763	3		24,550–25,050		(Yang et al., 2021)
	*qRV-9*	mk2124–mk2127	9		20,850–21,150		
	*qRV-11*	mk2505–mk2508	11		28,150–28,550		
Root Fresh Weight	*qRSA-3*	mk758–mk763	3		24,550–25,050		(Yang et al., 2021)
	*rrsf3*	G51-RM231	3	48			(Mu et al., 2003)
	*rrsf12*	RM252-RM270	12	4			
	*rrsf2*	RM341-RM208	2	22			
	*rrsf6*	R1962-G1314	6	10			
Number of Roots	*rn1a*	C161A-RM243	1	44			(Mu et al., 2003)
	*rn1b*	RM243-RM259	1	0			
	*rn2*	RM208-RM48	2	0			
	*rn7*	OSR22-RM11	7	6			
	*rn11a*	C950-C6	11	2			
	*rn5*	RM161-R521	5	12			
	*rn6*	RM276-RM253	6	2			
	*rn4*	RM348-RM349	4	0			
	*rn11b*	RM287-RM209	11	0			
	*rn11c*	G181-G320	11	26			
	*qRNO8*	C8M27	8	200			(Ranaivo et al., 2022)
	*qPef9*	C9M16	9	96			
Root dry weight	*rdw1a*	C813-C955	1	0			(Mu et al., 2003)
	*rdw1b*	RM5-RM302	1	0			
	*rdw3*	RM60-C814	3	28			
	*rdw5b*	G1458-C246	5	4			
	*rdw7*	RM47-RM172	7	0			
	*rdw9*	R79-R2638	9	0			
	*rdw11b*	RM224-G181	11	0			
	*rdw2*	R712-G21	2	16			
	*rdw5a*	R566-R2289	5	4			
	*rdw11a*	C6-OSR1	11	12			
	*rdw11b*	RM224-G181	11	0			
	*rdw12*	RM101-RM260	12	12			
	*qRDW_3.1_*	id3001701-id3008333	3	3.2–24.5			(Singh et al., 2017)
	*qRDWNS1.2*		1		41096.834	Varying N concentrations	Phan et al., 2023
	*qRDWNS2.1*		2		23177.834		
	*qRDWNS8.2*		8		27640.269		
	*qRDWNS10.1*		10		14391.386		
	*qRDWNS1.1*		1		29517.723		
	*qRDWNS4.1*		4		19933.152		
	*qRDWNS8.1*		8		27602.390		
L-type density on crown root (LDC)	*qLDC5*	S05_27313585	5		26276.202	Phosphorus deficiency	(Dinh et al., 2023)
S-type density on crown root (SDC)	*qSDC1*	S01_29957378	1		29269.714		
S-type density on L-type (SDL)	*qSDL9*	S09_8741627	9		8182.160		
Single S-type length on L-type (SLL)	*qSLL1*	S01_41579214	1		40879.329		
Early vigor	*qEV_3.1_*	id3001701-id3008333	3	3.2–24.5			(Singh et al., 2017)
	*qEV_3.2_*	id3010173-id3013447	3	11.3–29.5			
	*qEV_4.1_*	id4012189-id4004461	4	16.2–35.3			
	*qEV_5.1_*	wd5002636-id5001470	5	2.5–19.5			
	*qEV_5.2_*	id5007323-id5013100	5	3–29.6			
	*qEV_6.1_*	ud6000218-id6007312	6	11.7–27.6			
Seedling vigour index	*qSVI11.1*	RM3701	11	46			(Barik et al., 2022)
	*qSVI8.1*	RM502	8	178			
	*qSVII2.1*	RM13335	2	8			
	*qSVII6.1*	RM103	6	90			
	*qSVII6.2*	RM3	6	190			
	*qSVII11.1*	RM441	11	348			
N use efficiency	*qNUEl2–1*	RM53–R1738	2	80.5		Low Nitrogen	(Wei et al., 2011)
	*qNUEl6*	R2749–R1952a	6	173.2		Low Nitrogen	
	*qNUEn1*	C86–C2340	1	40.9		Optimum N	
	*qNUEn2–1*	RM53–R1738	2	84.5		Optimum N	
	*qNUEl2–2*	RZ599–RM53	2	62.5		Low Nitrogen	
	*qNUEl7–1*	RZ471–RG678	7	65.9		Low Nitrogen	
	*qNUEl7–2*	R1440–C1023	7	73.8		Low Nitrogen	
	*qNUEl11*	R3203–RM20a	11	165.7		Low N	
	*qNUEn2–2*	RM53–R1738	2	80.5		Optimum N	
Agronomic Nitrogen use efficiency		RM433–RM230	8	118.21			(Nguyen et al., 2016)
Physiological Nitrogen use efficiency		RM321–RM409	9	43.21			
		RM453–RM247	2	32.1			
Nitrogen uptake ability	*qNUP2.1*		2		36017.98–36777.82		(Zhou et al., 2017)
	*qNUP3.1*		3		25056.24–25069.45		
	*qNUP6.1*		6		7814.67–9668.4		
	*qNUP8.1*		8		2797.91–3336.1		
	*qNUP10.1*		10		22335.39–22517.95		
	*qNUP11.1*		11		19120.16–19494.14		
	*qNUP11.2*		11		25559.19–26317.7		
Nitrogen use efficiency	*qNUE2.1*		2		31531.95–32386.1		
	*qNUE4.1*		4		23285.5–23315.5		
	*qNUE6.1*		6		6517.44–6942.38		
	*qNUE6.2*		6		9668.4–9927.7		
	*qNUE10.1*		10		17355.11–17376.7		
	*qNUE10.2*		10		20364.8–20798.4		
P uptake at maturity	*qPUP1*	BIN46-BIN47	1	36.1			(Wang et al., 2014)
	*qPUP7*	BIN1007-BIN1008	7	55			
	*qPUP10*	BIN1348-BIN1349	10	37.7			
P Harvest Index	*qPHI1*	BIN59-BIN60	1	63.4			
	*qPHI2*	BIN310-BIN311	2	99			
	*qPHI6*	BIN838-BIN839	6	9			
	*qPHI11*	BIN1392-BIN1393	11	2.2			
Grain P use efficiency	*qgPUE4*	BIN680-BIN681	4	106.6			
Straw P use efficiency	*qstrPUE1–1*	BIN60-BIN61	1	63.9			
	*qstrPUE1–2*	BIN177-BIN178	1	150.8			
	*qstrPUE2*	BIN302-BIN303	2	79.6			
P use efficiency for biomass	*qPUEb2*	BIN253-BIN254	2	39.5			
P use efficiency for grain yield	*qPUEg1*	BIN143-BIN144	1	135.9			
	*qPUEg2*	BIN302-BIN303	2	79.6			
	*qPUEg6*	BIN946-BIN947	6	112.7			
	*qPUEg11*	BIN1395-BIN1396	11	5.2			
	*qPUEg12*	BIN1612-BIN1613	12	100.1			
P Translocation	*qPT2*	BIN294-BIN295	2	75.7			
	*qPT5*	BIN709-BIN710	5	13.9			
	*qPT8*	BIN1130-BIN1131	8	56.3			
P Translocation efficiency	*qPTE1–1*	BIN33-BIN34	1	30.1			

Further, breeders are empowered to genotype more correctly due to the improvement in sequencing quality and decline in cost (Varshney et al., 2021). Rice has a smaller genome among cereals, so genomic data may be quickly processed and interpreted using various computational tools. Numerous GWAS studies have recently discovered QTLs and candidate genes for traits required for the LI system (**Table 2**). Unfortunately, these studies have yet to employ phenotyping under the LI method. Hence, phenotyping data for existing LI systems should be included better to predict the marker-trait association (MTA) vital for LI systems. Apart from this, breeders can quickly uncover novel MTA using genotyping data, and the findings may be applied to upcoming research projects. Molecular markers and genomic selection (GS) can be employed in rice breeding to select variations linked with desirable traits discovered by GWAS (Chen et al., 2019). Since GWAS may indicate prospective regions or alleles for alteration, GWAS data can also be employed in crop improvement using CRISPR-Cas9-based gene editing (Tsakirpaloglou et al., 2023). However, GWAS approaches present considerable statistical challenges due to low power in detecting rare variants. Researchers use numerous techniques to address this problem, including enormous sample sizes or multiple biparental cross populations (Huang and Han, 2014).

**Table 2:** Reported GWAS studies in rice for traits related to LI systems.

Trait	Accessions	Number of SNPs	Number of QTLs	No. of genes	Reference
Sheath blight resistance	299	44,000	2		(Chen et al., 2019)
	563	2,977,750	27		(Zhang et al., 2017)
	417	3.4 million	18		(Li et al., 2022)
	259	2,888,332		653	(Wang et al., 2021a)
Bakanae resistance	143	542,333	1	34	(Lee et al., 2022)
	138	166,418	2	129	(Volante et al., 2017)
Bacterial blight resistance	172	317,894	12		(Zhang et al., 2017)
	259	2,888,332		109	(Shu et al., 2021)
	340	3K	11		(Lu et al., 2021b)
Blast resistance	355	5291		2341	(Lu et al., 2019)
	413	3835	16	74	(Zhu et al., 2016)
	584	700,000	27		(Liu et al., 2020)
Bacterial leaf streak resistance	236	176,820	12	29	(Sattayachiti et al., 2020)
Rice black-streaked dwarf virus resistance	420	44,000	13		(Feng et al., 2019)
Root-knot nematode resistance	332	44,100	11	493	(Dimkpa et al., 2016)
N deficiency tolerance	230	3,180,471	5	24	(Li et al., 2022)
N use efficiency related traits	267	151,202	12	18	(Xin et al., 2021)
P use efficiency related traits	413	44K	6	12	(Wissuwa et al., 2015)
P use efficiency related traits	157	25,971	18	85	(To et al., 2020)
Early root vigor	307	223.6K	6		(Wang et al., 2018a)
Rice early seedling vigor related traits	200	161,657	224	1609	(Zeng et al., 2021)
	244	7,098	43		(Thapa and Septiningsih, 2021)
Root length	178	403,559,	4	1	(Zhao et al., 2021a)

Additionally, for a better understanding of gene function and a lower error rate, the post-GWAS study should incorporate multiple in-silico analyses leveraging bioinformatic tools. For instance, further research should be performed to exclude misleading genes other than causal genes inside an LD block containing significant SNPs (Tibbs Cortes et al., 2021). Additionally, SNP-based GWAS may yield spurious conclusions. For this reason, many researchers choose GWAS based on haplotypes. Haplotypes, for instance, can circumvent issues with SNP-based GWAS studies, such as linkage drag, biallelic nature, and the absence of uncommon alleles (Bhat et al., 2021). A haplotype is a group of alleles inherited with a low chance of recent recombination for several polymorphisms on the same chromosome (Stram, 2017). SNP markers are less effective and potent than haplotype markers, and recent research has revealed improved haplotype-based GWAS and GS in plants. Considering these advantages, it is possible to find valuable markers, genes, and QTLs for the rice LI system using haplotype- or SNP-based GWAS.

### 4.7 Genomic selection for LI systems

Genomic selection (GS) significantly shortens breeding cycles and improves selection efficiency in a less expensive way. While association mapping can identify many QTLs and candidate genes, it cannot identify genomic regions contributing to small effects. Therefore, GS has a greater potential to capture all the effects found in the genomic information than association mapping. Additionally, the power and linkage drag issues of the GWAS technique are resolved by the GS method. Similarly, GS is more effective than conventional marker-assisted selection (MAS) (Lorenz et al., 2011). The first consideration to starting genomic selection is the appropriate size for the training population and the testing population. Plant breeders, as opposed to animal breeders, can use a small training population to implement genomic selection in cereals (Lorenz et al., 2011). Due to this, GS in rice has vast potential for crop improvement for LI systems. In addition, the genetic relationship between the training and testing population also plays a vital role in prediction accuracy. For instance, more genetically related lines in the training population set might enhance prediction accuracy (Liu et al., 2018b). Also, the distance between the training population and the testing population can lower prediction ability across the breeding cycle selection (Robertsen et al., 2019). Therefore, the training and testing population should be maintained carefully, covering all these issues.

The LI system should also cover many criteria along with stability in a diverse environment. Crops under the LI system must endure variable environmental conditions through their genetic ability to survive. So, studying GxE interaction will be advantageous for developing LI system-adapted cultivars. Moreover, GS has the capacity to add GxE interaction into the prediction model (Crossa et al., 2017), which is particularly helpful in predicting the impact of the environment on genotype stability. Furthermore, the incorporation of GxE interaction with the prediction model increased prediction accuracy in many kinds of cereal. For instance, the genomic prediction model addressing GxE interaction increased selection accuracy in bread wheat, maize, and legumes (Burgueño et al., 2012; Cuevas et al., 2016; Crossa et al., 2017; Jarquín et al., 2017). Another crucial point for the LI system is the selection of multiple traits. Since the desired response of cultivar in a LI system relies on several traits, a multi-traits-based selection approach will be perfect for LI systems. Researchers reported increased efficiency in genomic prediction through a multi-trait random regression model for daily water usage and shoot biomass in rice (Baba et al., 2020). Multiple traits-based GS performs better amid the unavailability of phenotypic data for every individual or trait (Jia and Jannink, 2012). Ben Hassen et al. (2018) showed that the multi-environment model enhances the prediction ability of heading dates, panicle weight, and nitrogen balance index under different water management conditions in rice. Furthermore, traits that show low heritability can be more effectively bred through a multi-trait-based genomic selection model (Xu et al., 2021). Since superior performance depends on several traits, it is advantageous to predict the performance of certain traits based on other traits. In this context, multi-trait GS can forecast the performance of traits that are incredibly challenging to phenotype from other related traits (Xu et al., 2021). Through the advancement of high-throughput techniques, components can be related to predicting the performance of certain traits. For example (Sun et al., 2017), studied the prediction accuracy of wheat grain yield from secondary traits such as canopy temperature and normalized difference vegetative index. Again, the inclusion of the selection index in the prediction model can also increase the efficacy in the prediction for suitable genotypes. For example, Wang et al. (2019) included a selection index in the GS model to better predict grain yield through auxiliary traits in rice.

### 4.8 Gene editing for LI systems

The recent advances in gene editing techniques have made gene editing a fast, safe, and reliable option for crop improvement. Since manipulation can be done within the target genome, the breeders save some time and effort. Besides, GWAS can identify novel genomic regions that can be modified through genome editing. In this context, CRISPR-Cas9-based techniques can advance the trait improvement process by targeting certain traits in an advanced line. Several genes/alleles or miRNAs have been identified as important for LI agriculture’s trait improvement (**Table 3**). Genome editing techniques can be applied to improve some of the traits by modifying these alleles. For increasing nitrogen use efficiency (NUE), transporter(s) related to each nutrient and genes regulating the desired ideotype should be targeted. Fortunately, some recent studies successfully integrated CRISPR-based genome editing for NUE in different crops. For instance, Karunarathne et al. (2022) demonstrated the scope of increasing NUE in barley by targeting the *ARE1* gene. Lu and Zhu (2017) showed the efficiency of base editing with the *NRT1.1B*-indica allele that contributes to the higher nitrate uptake in indica rice (Hu et al., 2015). Similarly, past studies documented that genetic manipulation could modify root development. For instance, *OsACS* mutants showed reduced lateral root growth under phosphorus deficiency, suggesting a scope of enhancing lateral root growth by overexpressing ethylene synthesis-related genes in rice (Lee et al., 2019). Kitomi et al. (2020) modified a homolog of *DRO1* (Deep rooting-1) to modify the root angle for increasing rice yield under saline conditions. Additionally, multiplex genome editing created a new opportunity to target several plant genes simultaneously. Since many disease-resistant traits are controlled by more than one gene, this multi-gene-based editing will be beneficial in developing rice cultivars resistant to multiple diseases. Although many genes have been pyramided for disease resistance in plants, the multiplex genome editing approach will be a quicker and more pragmatic solution. For example, researchers documented rice blast and bacterial blight-resistant sterile lines through multiplex genome editing by adding specific mutations into *TMS5, Pi21*, and *Xa13* genes (Li et al., 2019a). Moreover, knocking out the genes related to susceptibility, enhancing the expression of resistance genes, and changing the interaction between the effector and target can be potential areas of modification through genome editing (Bisht et al., 2019). One study showed that a single nucleotide polymorphism site in *eIF4G* is responsible for resistance against Rice Tungro Spherical Virus (Lee et al., 2010). Such sites can be easily edited by CRISPR-based genome editing techniques. Additionally, gene-edited products can remove the dissatisfaction associated with GM (genetically modified) crops since Cas9 can be removed from plants through natural segregation.

**Table 3:** Genes identified for traits related to LI systems in rice.

Genes/proteins	Description	Function	References
*OsPTR6*	Peptide transporter 6	Overexpression increases plant height and biomass under a certain aluminum amount	(Fan et al., 2014)
*OsGS1;2*	Glutamate synthetase	Increase NUE and grain yield	(Chen et al., 2023b)
*OsNPF3.1*	Nitrate peptide transporter family gene	Improve nitrogen utilization efficiency	(Hang et al., 2024)
*OsHHO3*	HRS1 Homolog 3	Negatively regulates NUE	(Liu et al., 2023)
*OsSPL14*	Squamosa promoter binding protein-like 14	Promotes suitable plant architecture for N acquisition	(Srikanth et al., 2016)
*Lw5*	Leaf width 5	Modulates NUE through plant architecture	(Zhu et al., 2020)
*TaGS1*	Glutamate Synthetase 1	Increase NUE	(Wu et al., 2021a)
*Ghd7*	Grain number, plant height, and heading date 7	Increase NUE by repressing *ARE1*	(Wang et al., 2021b)
*dep1*	dense and erect panicle 1	Higher N utilization efficiency	(Huang et al., 2022)
*OsNPF3.1*	NITRATE TRANSPORTER 1/PEPTIDE TRANSPORTER	Affects NUE in rice	(Yang et al., 2023)
*OsNLP1*	NIN-LIKE PROTEIN 1	Increase NUE	(Alfatih et al., 2020)
*OsATG8b*	Autophagy-related genes	Nitrogen stress tolerance	(Zhen et al., 2019)
*OsNPF7.7*	nitrate and peptide transporters family	Increase NUE	(Huang et al., 2018)
*PP2C9*	Protein phosphatase	Increase nitrate reductase activity	(Waqas et al., 2018)
*OsCIPK2*	Calcineurin B-like interacting protein kinase 2	Increase NUE	(Khan et al., 2019)
*OsNPF7.6*	nitrate transporter1/peptide transporter family	Increase NUE	(Zhang et al., 2022a)
*qNGR9*	heterotrimeric G protein complex	Increase NUE	(Sun et al., 2014)
*miR169o*		Increase NUE and susceptibility of bacterial blight	(Yu et al., 2018)
*OsPTR9*	Peptide transporter 9	Increase NUE	(Fang et al., 2013)
*OsNRT2.1*	NITRATE TRANSPORTER	Increase NUE	(Chen et al., 2016)
*OsNLP4*	rice NIN-like protein 4	Increase NUE	(Wu et al., 2021b)
*OsENOD93–1*	Rice early nodulin gene		(Bi et al., 2009)
*AlaAT*	Barley alanine aminotransferase	Increase NUE	(Shrawat et al., 2008)
*OsNLP4-OsNiR*	Cascade of NIN-like protein and genes encoding nitrate reductase	Increase NUE	(Yu et al., 2021)
*OsNLP3*	Rice NIN-like protein 3	Increase NUE	(Zhang et al., 2022b)
*gs3*	Glutamate synthetase 3 allele	Increase NUE	(Yoon et al., 2022)
*ARE1*	abc1–1 repressor1	Decreases NUE	(Wang et al., 2018b)
*OsNR2*	Rice nitrate reductase 2	Increase NUE	(Gao et al., 2019)
*MYB61*	Myeloblastosis genes	Regulated by GRF4 and increase NUE	(Gao et al., 2020)
*NRT1.1B*	Indica nitrate transporter	Involved with root microbiome and nitrogen use	(Zhang et al., 2019)
*OsAMT1;2 and* *OsGOGAT1*	Ammonium transporter 1;2 and Glutamate synthetase 1	Concurrent activation enhance tolerance to low N	(Lee et al., 2020)
*OsGLP1*	Rice germin-like protein1	Increase plant height and disease resistance	(Banerjee and Maiti, 2010)
*OsERF83*	rice ethylene response factor	Blast resistance	(Tezuka et al., 2019)
*Xa23*	Executor R gene from wild rice	Bacterial blight resistance	(Wang et al., 2015)
*OsGRDP1.*	glycine-rich domain protein	Regulate cell death and disease resistance	(Zhao et al., 2021b)
*OsWRKY76*		Overexpression increases cold tolerance but decrease blast resistance	(Yokotani et al., 2013)
*OsCPK10*	Calcium-dependent protein kinase	Increase blast resistance and drought tolerance	(Bundó and Coca, 2016)
*OsWRKY76*		Increase blast resistance	(Chujo et al., 2014)
*OsPR10a*	Pathogenesis-Related Protein	disease resistance	(Huang et al., 2016)
*OsCIPK30*	calcineurin B-like proteins	Rice stripe virus tolerance	(Liu et al., 2017)
*miR396-OsGRFs*	Rice micro-RNA and growth regulation factors	Growth and disease resistance	(Chandran et al., 2019)
*OsMPK15*	Mitogen-activated protein kinase 15	negatively regulate the disease resistance	(Hong et al., 2019)
*OsDCL1a*	DICER-Like (DCL) ribonuclease	negatively regulate the disease resistance	(Salvador-Guirao et al., 2019)
*OsNramp6*	Natural resistance-associated macrophage proteins	Disease resistance	(Peris-Peris et al., 2017)
*Cu/Zn-Superoxidase Dismutase1, Cu/Zn-Superoxidase Dismutase and Os11g097,80*	Target genes of miR398b	Disease resistance	(Li et al., 2019b)
*OsOSM1*	Rice osmotin gene	Sheath blight resistance	(Xue et al., 2016)
*WRKY45*		Bacterial blight and blast resistance	(Goto et al., 2015)
*OsCPK4*	Calcium-dependent protein kinase	blast resistance	(Bundó and Coca, 2016)
*OsACL-A2*	ATP-citrate lyases	negatively regulate the disease resistance	(Ruan et al., 2019)
*OsMESL*	methyl esterase-like	Broad-spectrum disease resistance	(Hu et al., 2021)
*OsPLDb1*	Phospholipase D	negatively regulate the disease resistance	(Yamaguchi et al., 2009)
*OsMADS26*	MADS-box transcription factors	negatively regulate the disease resistance and drought tolerance	(Khong et al., 2015)
*OsMPK3*	Mitogen-activated protein kinase 3	striped stem borer defense response	(Wang et al., 2013)
*OsbHLH057*	basic/helix-loop-helix	Enhance the disease resistance and drought tolerance by modulating the expression of Os2H16	(Liu et al., 2022)
*OsMPK6*	Mitogen-activated protein kinase	negatively regulate the blast disease resistance	(Yuan et al., 2007)
*WRKY30*		disease resistance	(Peng et al., 2012)
*OsACDR1*	Oryza sativa accelerated cell death and resistance 1	disease resistance	(Kim et al., 2009)
*ONAC066*	NAM, ATAF, and CUC (NAC) transcription factor	disease resistance	(Liu et al., 2018a)
*OsSnRK1a*	Sucrose non-fermenting-1-related protein kinase-1	Broad-spectrum disease resistance	(Filipe et al., 2018)
*OsPT8*	Phosphate Transporter Protein	negatively regulate the disease resistance	(Dong et al., 2019)
*OsHKT 2;1*	sodium transporter	Potassium use efficiency	(Hartley et al., 2020)
*OsHAK16p: WOX11*	WUSCHEL-related homeobox gene with OsHAK16p promoter	Potassium use efficiency	(Chen et al., 2015)
*OsJAZ9*	JASMONATE ZIM 9	Improves K deficiency tolerance	(Singh et al., 2020)
*OsPTF1*	rice Pi starvation induced transcription factor 1	Improves tolerance to Pi starvation	(Yi et al., 2005)
*OsIPMS1*	isopropylmalate synthase	Improve seed vigor	(He et al., 2019)
*OsHIPL1*	hedgehog-interacting protein-like 1	Improve seed vigor	(He et al., 2022)

## 5 Prospects of organic rice production

The safe food movement made organic farming (OF) a practical alternative to crop production, as people became increasingly cautious about their food choices. As a corollary, growers across the world are becoming more interested in organic rice. Like the low-input systems, varieties for organic systems are also being tested in the conventional systems. However, a combination of evolutionary and participatory breeding will be helpful to develop superior varieties for both organic and low-input systems. Many farmers choose to prefer the standard high-input methods over the organic systems since the yield of OF is significantly lower than that of conventional systems. However, the low yield of organic rice is offset by a better benefit-cost ratio, which compensates for the reduced yield. For instance, research done in Thailand showed that despite the poor yield, OF had higher economic benefits due to the cheap production cost and higher market value (Arunrat et al., 2022). Furthermore, organic rice may be a financially feasible alternative for farmers in developing nations that have limited resources since organic amendments combined with management practices can establish a profitable organic farm (Mishra et al., 2018). In addition to the health advantages, organic rice farming has a positive impact on the environment. Soil organic carbon and soil carbon sequestration, for instance, will be less affected by organic farming in the context of climate change (Arunrat et al., 2021). Further, sustainable crop production methods should safeguard the complex biological interactions between many organisms within a given environment. Fortunately, rice fields’ biodiversity may be preserved and increased via the use of organic agriculture practices (Katayama et al., 2019). Nevertheless, due to the current interconnectedness of other stakeholders, only OF practices cannot maintain an entirely sustainable system. Accordingly, a combination of OF and other agricultural techniques is required for sustainability (Reganold and Wachter, 2016). For instance, an efficient organic fertilization system combined with crop rotation is the most effective method for variable climatic conditions (Arnés et al., 2013).

## 6 Hunt for microbiome-friendly release

Nature has endowed wild plants with a unique association capability with microbes, and this association benefits crops in many ways. However, the domestication of wild plants adversely affected the beneficial plant-microbe interaction of wild species (Pérez-Jaramillo et al., 2016). Modern plant breeding should emphasize utilizing maximum symbiosis from crop cultivars and select materials hospitable to soil microbial composition. In this context, using beneficial microbes from wild plants and seed endophytic bacteria can be a positive way of achieving better sustainability (L’Hoir and Duponnois, 2021).

Non-leguminous crops like rice can be improved through the exploitation of arbuscular mycorrhizal associations. Microbes help plants in many ways, including adverse effects on disease-forming pathogens, abiotic stress tolerance, and nutrient uptake efficiency (Bulgarelli et al., 2013). In particular, Arbuscular mycorrhizal fungi (AMF) helps plants to maintain proper growth under P deficiency (Harrison et al., 2002; Smith et al., 2003). Arbuscular mycorrhizal symbiosis can also be a potential way of improving rice yield and stress tolerance (Mbodj et al., 2018). Previous studies have reported the transfer of nutrients such as N, P, S, and Fe from mycorrhiza-like fungi to plants. A study showed that *Piriformospora indica* transfers Fe to rice (Verma et al., 2022). Very few efforts have been made to identify genetic factors controlling the root microbiome of rice under stress conditions. A recent study identified 10 SNPs and ten candidate genes related to the root microbiome of rice under drought stress. They identified more major fungal clusters. One of the fungal clusters, Pleosporales, is a crucial member of the rice seed microbiome (Eyre et al., 2019) and has previously been shown to improve plant growth and nitrogen content (Vergara et al., 2019). Another study identified 23 putative QTLs related to root colonization in rice (Davidson et al., 2019). In addition, substituting *OsCERK1DY* allele from wild rice to an indica variety ZH11 improved the phosphorus uptake by increasing AMF colonization (Huang et al., 2020). One study also found four QTLs related to associative N2 fixation in rice (Wu et al., 1995; Kaeppler et al., 2000).

Though soil is the primary determinant of microbial composition, plant species also play a crucial role in determining microbiome. Researchers have documented the effect of various genotypes in maintaining microbiome composition (Bergelson et al., 2019; Schmid et al., 2019; Jacoby et al., 2020). For example, Lundberg et al. (2012) documented that different *Arabidopsis* accessions have distinct microbiome compositions. In rice, researchers also found genotypic variation in microbiome composition. For example, root endophytes like *Azoarcus* spp. and *Acremonium* preferentially prefer wild species for colonization (Engelhard et al., 2000). Researchers found differences in nitrogen derived from air (ndfa) by comparing diverse rice lines (Shrestha and Ladha, 1996). Traditional varieties with high ndfa value showed high associative fixation and sustained 12 months on N-free medium (Rolfe et al., 1997).

For this reason, screening genotypes for microbiome friendliness should be considered. Two things need to be appropriately considered for comparing the genotypes’ feasibility in plant-microbiome interactions: root morphology and root exudates.

Root morphology can influence microbiome composition by affecting nutrient availability. For example, longer roots reduce microbial biomass by decreasing nitrogen availability (Pérez-Jaramillo et al., 2017; Wan et al., 2021). Root traits such as root length, root hair, and root branching patterns are essential in determining microbial diversity (Eisenhauer et al., 2017; Robertson-Albertyn et al., 2017; Wang et al., 2017). Previous studies reported that roots having less root diameter attract more microbes (Szoboszlay et al., 2015; Wang et al., 2020b). Bacteria also can modify root traits by exerting certain phytohormones (Grover et al., 2021). AMF preferred larger lateral roots in rice for colonization (Gutjahr et al., 2009).

Further, root exudates are one of the prime factors for studying the rhizosphere. Metabolomics is the perfect means of characterizing rhizosphere based on metabolites. Metabolites can facilitate communication between beneficial microbes for adapting to adverse conditions or protecting plants against pathogens (Jacoby et al., 2020). Root exudates create a favorable environment for microbes by providing a proper nutritional source (Sasse et al., 2018). In addition, utilizing ‘holo-omic’ and ‘exometabolomics’ in the breeding pipeline can decipher useful plant-microbe interactions (Sasse et al., 2018; Nerva et al., 2022).

LI systems can be an effective solution for fostering and maintaining healthy associations between microorganisms. In low-input systems, management techniques and landscape variation impact the soil microbiota and enzyme activity (Wickings et al., 2016). However, the judicious application of different management approaches will determine the extent of the association.

## 7 Integrated breeding approaches for LI

**Figure 4:**
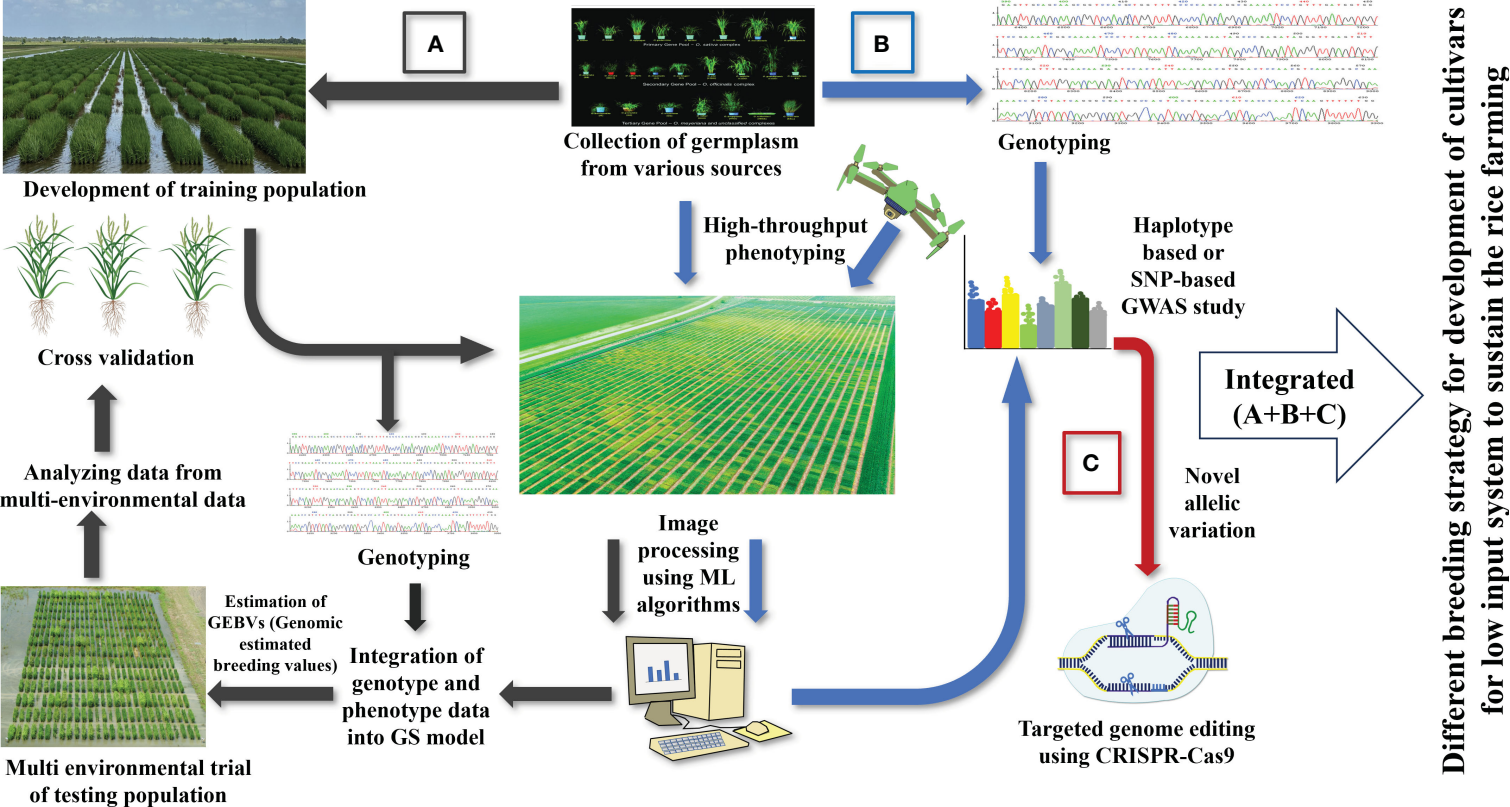
Various breeding strategies for developing rice cultivars for low input system: **(A)** Genomic selection approach to rapidly develop rice cultivars for LI systems, **(B)** genome wide association study for identifying QTLs, genes, and allelic variation important for LI systems, **(C)** CRISPR-based gene editing tool to create desired modifications.

A shift to LI agriculture would reduce pollution, reward farmers financially, and protect the environment. The current practice of developing high-yielding varieties fails to consider low-input cultivars (Vanloqueren and Baret, 2008). So, an integrative breeding approach should be designed to cover every facet of sustainable agriculture (**Figure 4**). Transforming modern agriculture to a low-input sustainable system requires new varieties that can thrive under extreme conditions. Varieties for the LI system should be equipped with multiple traits, making it a cumbersome task. Fortunately, the advent of modern technologies can be incorporated in the breeding program to reduce the timeframe required for developing a variety of LI systems. The first step will be exploring germplasms for enhanced resource use efficiency and multiple stress resistance. Since most of our materials are adapted to high input conditions, we must screen many materials for starting any breeding program for LI systems. Recent phenomics developments will help breeders rapidly screen several germplasms to identify the perfect candidate that can be included in the crossing program. The flexibility of machine learning algorithms can infer multiple parameters just by analyzing field images. Important traits such as early vigor and nutrient use efficiency can be tested within a shorter time with adjustment in image processing pipeline.

Further, genetic factors behind traits like early vigor, nutrient use efficiency, and stress resistance have already been explored. For instance, genes controlling nitrogen assimilation and utilizations like *OsNPF3.1, OsNPF7.1, OsNRT2.1* can be targeted for better nitrogen use efficiency. Rice diseases like blast, and bacterial leaf blight are vastly studied, and multiple genes are identified, for instance, *OsCPK4* for blast resistance, *Xa23* for bacterial leaf blight resistance, *OsOSN1* for sheath blight resistance. Pyramiding multiple genes will be an excellent way to develop advanced lines for low-input agriculture. Another critical aspect of sustainable agriculture is root traits. The modern phenomics pipeline has lessened the difficulty in phenotyping root traits. Traits such as root length, diameter, and hair density would have been complex to study without phenomics platforms. Incorporation of root phenomics in the screening program will efficiently identify good lines for resource use efficiency. Various metabolomics studies can be done simultaneously to identify microbe-friendly genotypes since the microbiome is crucial for maintaining sustainability. Also, we must explore new genes, QTLs, and markers under low-input conditions by utilizing cheaper genotyping data. A genome-wide association study should be conducted for all the essential traits for identifying new markers and genomic regions associated with desired traits. Searching for these genes or alleles to introgress into existing lines will be beneficial for developing an ideal variety for the LI system. In addition, identified genes can be validated easily by incorporating genome editing tools. This validation step will increase the success rate before starting to pyramid multiple genes. Since genotyping has become cheaper than phenotyping, genomic selection will be a handy tool in any breeding program. While training the genomic selection model, we should incorporate all the relevant traits for the LI system. Finally, genomic selection will be helpful in rapidly testing advanced lines.

## 8 Conclusion and future perspectives

We must modify our conventional agricultural methods into resource-efficient systems to attain sustainability in agricultural practices. In this context, modern development in genomic research can facilitate rice breeding for low-input systems. A successful rice breeding program for a low-input system will require fast identification of traits that promote resource use efficiency, better protection against pathogens, and incorporating these traits into one cultivar. Utilizing all modern tools throughout the process will increase selection efficiency and reduce the number of breeding cycles required. Since rice is cultivated across a range of environments, breeding practices should consider precise estimation of genotype-environment interaction. Recent developments in environmental modeling have created new opportunities to predict the performance of certain genotypes by including multiple environmental covariates in the prediction model. Besides, developing countries may face adverse environmental conditions due to climate change. Testing the performance of LI rice in stressed environments will provide better insights into its suitability in developing countries.

Initially, we must identify early vigorous rice genotypes that can compete with weeds. Tolerance to multiple stress factors will also play a vital role. Moreover, phenotyping for root traits will be the most effective way to search for nutrient use efficiency. Genes related to nitrogen transportation and assimilation, like *OsNLP4, OsNPF7.7, and gs3* allele, can be advantageous. In general, nutrient use efficiency traits are polygenic in nature. QTLs associated with nitrogen, phosphorus, and potassium use efficiency have already been mapped in rice. For early vigor *OsIPMS1, OsHIPL1* genes can be utilized. Root traits such as root lengths, diameter, and surface area are essential for nutrient uptake. Fortunately, many high-throughput platforms have been developed to facilitate these phenotyping processes. Although people are using many of these platforms regularly, we should be careful to limit the misinterpretation of the data. In addition, breeders should cross-validate their results for better accuracy. Besides, researchers should continuously improve machine learning and deep learning algorithms for better accuracy in high-throughput phenotyping. Also, cross-validation should be a routine task associated with all these methods.

Further, cheaper NGS data can be used in genome-wide association mapping for identifying genes, QTLs, and markers for multiple traits. Proper selection of genotyping platforms, marker system, and accurate phenotyping will determine the efficiency of this method. Besides, the genomic selection model can help evaluate many lines for multiple traits in a shorter time. Also, the ability to use a multi-trait model or incorporation of phenomics data made GS a powerful tool in breeding for low-input systems. In predictive breeding, minimizing the error rate is a vital step. For this reason, data analysts should carefully manage the statistical packages to reduce the error rate. Breeders should also focus on newer statistical models that can generate more accurate predictions of their dataset.

Furthermore, genome editing can also help modify target genes for nutrient use efficiency or stress tolerance. However, we must be careful with gene editing tools and transgenic approaches since these techniques have acceptability issues. Gene editing can reduce the time for introducing the desired variation, but transforming plants for editing often becomes difficult. Fortunately, people are working on DNA and tissue culture-free methods for better efficiency in genome editing (Altpeter et al., 2016). Sometimes, getting a homozygous mutation becomes complex with the increased number of target genes in multiplex editing. More work on multiplex editing can render this a helpful approach for targeting multiple genes for low-input systems.

For developing a sustainable farming system, the soil microbiome must be taken care of. Microbes not only improve soil health but also improve nutrient availability to plants. For this reason, all lines should be checked thoroughly for microbiome compatibility. Metabolomic and phenomics studies can be used to study the genotypic effect on soil microbiome. Attaining sustainability relies on covering every facet of environmental safety. Hence, breeding rice for the LI system should be carefully crafted to ensure maximum protection of all natural resources.

## Author contributions

SJ: Conceptualization, Data curation, Formal analysis, Methodology, Software, Visualization, Writing – original draft, Writing – review & editing. GS: Methodology, Writing – review & editing, Visualization. AP: Visualization, Writing – review & editing. LT: Writing – review & editing. ES: Writing – review & editing. ST: Conceptualization, Funding acquisition, Methodology, Supervision, Writing – review & editing, Resources.
